# Prognostic Significance of Cytoplasmic SPNS2 Expression in Patients with Oral Squamous Cell Carcinoma

**DOI:** 10.3390/medicina57020164

**Published:** 2021-02-12

**Authors:** Jeng-Wei Lu, Yen-Shuo Tseng, Yu-Sheng Lo, Yueh-Min Lin, Chung-Min Yeh, Shu-Hui Lin

**Affiliations:** 1 Department of Biological Sciences, National University of Singapore, Singapore 117543, Singapore; jengweilu@gmail.com; 2 Department of Dermatology, Changhua Christian Hospital, Changhua 500, Taiwan; 125108@cch.org.tw; 3 Department of Oral Cancer Research Center, Changhua Christian Hospital, Changhua 500, Taiwan; 165304@cch.org.tw; 4 School of Medicine, Chung Shan Medical University, Taichung 402, Taiwan; 93668@cch.org.tw; 5 Department of Surgical Pathology, Changhua Christian Hospital, Changhua 500, Taiwan; 28935@cch.org.tw; 6 Department of Medical Technology, Jen-Teh Junior College of Medicine, Nursing and Management, Miaoli 356, Taiwan; 7 Department of Medical Laboratory Science and Biotechnology, Central Taiwan University of Science and Technology, Taichung 406, Taiwan

**Keywords:** SPNS2, immunohistochemistry, tissue microarray, oral squamous cell carcinoma, prognosis

## Abstract

*Background and Objectives:* Oral squamous cell carcinoma (OSCC) is a malignant disease with a particularly high incidence in Taiwan. Our objective in this study was to elucidate the involvement of sphingolipid transporter 2 (SPNS2) expression and SPNS2 protein expression in the clinicopathological indexes and the clinical outcomes of OSCC patients. *Materials and Methods:* Immunohistochemistry analysis was performed for SPNS2 protein expression in samples from 264 cases of OSCC. Correlations of SPNS2 expression with clinicopathological variables and patient survival were analyzed. *Results:* Our results revealed that the cytoplasmic protein expression of SPNS2 in OSCC tissue specimens was lower than in normal tissue specimens. Negative cytoplasmic protein expression of SPNS2 was significantly correlated with T status and stage. Kaplan–Meier survival curve analysis revealed that negative cytoplasmic SPNS2 expression was predictive of poorer overall survival of OSCC patients in stage III/IV. We also determined that low SPNS2 expression was an independent prognostic factor related to overall survival among OSCC patients in stage III/IV from univariate Cox proportional hazard models. Multivariate Cox proportional hazard models revealed that cytoplasmic SPNS2 expression, T status, lymph node metastasis, and histological grade were independent prognostic factors for survival. *Conclusions:* Overall, this study determined that SPNS2 protein may be a useful prognostic marker for OSCC patients and potential therapeutic target for OSCC treatment.

## 1. Introduction

Oral squamous cell carcinoma (OSCC) is the most common form of malignant head and neck squamous cell carcinoma (HNSCC), with most cases originating in the oral cavity [1,2]. OSCC is considered an aggressive tumor due to its high morbidity, high mortality, clinical behavior, high local recurrence rate, and high likelihood of regional and/or distant metastasis [3]. OSCC incidence has been linked to the consumption of alcohol and/or tobacco, human papillomavirus (HPV) infection, and deficiencies of vitamins A, C, E, folic acid, zinc, and selenium [4,5,6,7,8,9]. Previous research has shown that HPV infections are both independent prognostic biomarkers for HNSCC [10]; however, details pertaining to the underlying mechanism(s) of OSCC tumorigenesis still need to be further investigated. The accumulation of genetic and epigenetic abnormalities in epithelial cells of the head and neck is also seen as a key factor in the initiation and progression of OSCC [11,12]. 

The low 5 year survival of patients with advanced OSCC can be attributed to late diagnosis and resistance to radiotherapy and chemotherapy. In addition, OSCC can seriously affect one’s quality of life and surgery often leaves patients grievously disfigured [1,13,14]. The incidence of OSCC is high in South and Southeast Asian countries, estimated at 12.7 cases per 100,000 [15,16]. Note that the incidence of OSCC among males in Taiwan has recently reached 29.2 cases per 100,000 individuals per year [16]. Several biomarkers that could potentially predict the prognosis of OSCC patients have recently been discovered; however, this research has not yet led to clinical applications [17]. The effective monitoring and treatment of OSCC patients depend on reliable prognostic markers of late-stage development [18,19]. 

Sphingosine-1-phosphate (S1P) is a lipid mediator derived from sphingosine and catalyzed by sphingosine kinase 1 (SPHK1) and sphingosine kinase 2 (SPHK2) [20,21]. S1P is a biologically active signaling molecule, which plays key roles in various physiological and pathological processes, such as immunity and cancer [22,23,24,25]. S1P promotes tumor growth by regulating angiogenesis, cell proliferation, migration, survival, and lymphangiogenesis in various forms of cancer [22,26,27]. Sphingolipid transporter 1 (SPNS1) and sphingolipid transporter 2 (SPNS2) are both members of the major facilitator superfamily (MFS). SPNS1 and vacuolar-type H+-ATPase (v-ATPase) regulate autolysosomal biogenesis via acidification associated with developmental senescence and survival [28]. It also appears that SPNS1 and L-leucine may alleviate autophagy dysfunction of Niemann-Pick type C in mouse models and humans [29]. SPNS2 has been shown to regulate the release and activity of S1P [30]. Spns2 has also been identified as causing for abnormal heart development, and Spns2 deficiency has been linked to early-onset progressive hearing loss [31,32]. In mice, Spns2 plays a critical role in inflammatory and autoimmune diseases, wherein Spns2 deletion appears to have a strong effect in alleviating the development of collagen-induced arthritis [33]. The SPNS2 sequence is highly homologous in vertebrates, including 95% homology between humans and mice as well as 72% homology between humans and zebrafish [34]. 

The knockdown of SPNS2 was shown to increase intracellular S1P levels in non-small-cell human lung cancer cells, thereby promoting cell migration and inhibiting apoptosis [35]. A notable reduction in pulmonary metastasis in Spns2-deficient mice was linked to the inability of tumor cells to establish metastatic foci [36]. By regulating the phosphorylation of the serine/threonine protein kinase (AKT) and the signal-regulated kinase (ERK) pathways, it appears that SPNS2 enhances the malignancy of colon cancer in terms of cell proliferation, migration, and invasion as well as inhibits apoptosis [37]. In acute myeloid leukemia, SPNS2 is an indicator of poor prognosis; however, the effects of SPNS2 could potentially be neutralized by allogeneic hematopoietic stem cell transplantation (allo-HSCT) [38]. At present, the correlation between SPNS2 protein expression and prognostic value in OSCC patients has yet to be conclusively determined. This study revealed that negative cytoplasmic SPNS2 protein expression was indicative of poor prognosis in OSCC patients in the III/IV stage.

## 2. Materials and Methods

### 2.1. OSCC Patients and Ethics Statement

This study recruited 264 OSCC patients from the Changhua Christian Hospital, Changhua, Taiwan (From January 2000 to December 2008). The main treatment was tumor removal and radical neck dissection, including post-operative irradiation as well as selective patients treated with 5-fluorouracil (5-FU) and cisplatin chemotherapy. This study was also approved by the Ethics Committee of the Changhua Christian Hospital and complied with the guidelines approved by the Institutional Review Board (IRB No. 171227, date of approval 23 January 2018). IRB gave approval to use formalin-fixed, paraffin-embedded (FFPE) decoding tissue microarray samples without informed consent.

### 2.2. Preparation and Evaluation of Tissue Microarrays

Pathological assessment based on standard OSCC tissues and then the tissue microarrays (TMAs) were prepared. All of the standard OSCC tissue slices were stained using hematoxylin and eosin (H&E), whereupon the morphology of the cancer based on representative lesions was confirmed by two senior pathologists. The pathological evaluation of tumor stages and histological differentiation was performed in accordance with protocols established by the American Joint Committee on Cancer (AJCC, 7th Edition) Tumor, Node, Metastasis (TNM) staging system and the Edmondson–Steiner grading system. The details for TNM grading: (1) T-class (Primary tumor)/Tx: primary tumor cannot be assessed, T0: no evidence of primary tumor, Tis: carcinoma in situ; intraepithelial or invasion of lamina propria, T1: tumor invades submucosa, T2: tumor invades muscularis propria, T3: tumor invades through muscularis propria into subserosa or into non-peritonealized pericolic or perirectal tissue, T4: tumor directly invades other organs or structures and/or perforates visceral peritoneum. (2) N-class (Regional lymph nodes)/Nx: regional lymph nodes cannot be assessed, N0: no regional lymph node metastasis, N1: metastasis in 1 to 3 regional lymph nodes, N2: metastasis in 4 or more regional lymph nodes. (3) M-class (Distant metastasis)/Mx: distant metastasis cannot be assessed, M0: no distant metastasis, M1: distant metastasis. The details for Edmondson–Steiner grading: (1) Well: well-differentiated, (2) Moderate: moderately differentiated, (3) Poor: poorly differentiated. For TMAs, typical OSCC tissues and adjacent epithelial tissues were harvested, included 264 primary OSCC samples. The samples were fixed using paraffin to perforate tissue cylinders (2 mm in diameter) to construct OSCC and adjacent TMAs by a homemade, semi-automated tissue array in the Department of Pathology at Changhua Christian Hospital [39]. 

### 2.3. Immunochemical Staining

Immunohistochemical staining was performed as previously described [39,40]. After deparaffinization and hydration using ethanol at various concentrations, the TMAs were subjected to antigen retrieval using 0.01 M citrate buffer (pH 6.0) in a microwave, and incubated sequentially in 3% H_2_O_2_ to inhibit endogenous peroxidase activity and then in 10% normal goat serum at 37 °C for 1 h. The TMAs were then mixed with solution containing polyclonal rabbit anti-human SPNS2 antibodies (Dilution 1:100×; Catalog number: NBP1-54345; Novus Biologicals, Littleton, CO, USA) at 4 °C overnight. The following day, the TMAs were assayed for immune complex using a LASB 2 kit (Dako, Carpinteria, CA, USA). After staining with aminoethyl carbazole for enzyme activity, the TMAs were again stained with hematoxylin. To determine the specificity of the SPNS2 antibodies for immunohistochemical staining, appropriate positive (colorectal cancer tissue as a known positive case) and negative (samples not incubated with the primary antibody) controls were included in the experiment. 

### 2.4. Immunochemistry Scoring 

In assessing the immunohistochemical staining results, the evaluation scores were evaluated by two blinded senior pathologists. Samples were classified as positive or negative based on the following scores describing SPNS2 protein expression: 0 (No staining), 1+ (Weak positive expression), 2+ (Moderately positive expression), and 3+ (Strongly positive expression). Finally, we were further classified into two groups: (1) negative group includes 0 (No staining); 1+ (Weak positive expression); (2) positive group includes 2+ (Moderately positive expression); and 3+ (Strongly positive expression) [39,40]. 

### 2.5. Statistical Analysis

All analyses were performed using Statistical Product and Service Solutions (SPSS, version 17) (SPSS, Inc., Chicago, IL, USA). Fisher’s exact test or the Chi-square test was used to detect the importance of the clinicopathological variables of cytoplasmic SPNS2 protein expression and OSCC. For the negative and positive cytoplasmic SPNS2 protein expression in stage III/IV OSCC patients, overall survival curves were derived using the Kaplan-Meier method, and cumulative survival rates were assessed using the log-rank test. Univariate and multivariate analysis was performed to confirm prognostic factors of OSCC using the Cox proportional hazard regression model [39,40,41]. Statistically significant results were defined by a *p* value of < 0.05. 

## 3. Results

### 3.1. Demographic and Clinical Characteristics of OSCC Patients

**Figure 1:**
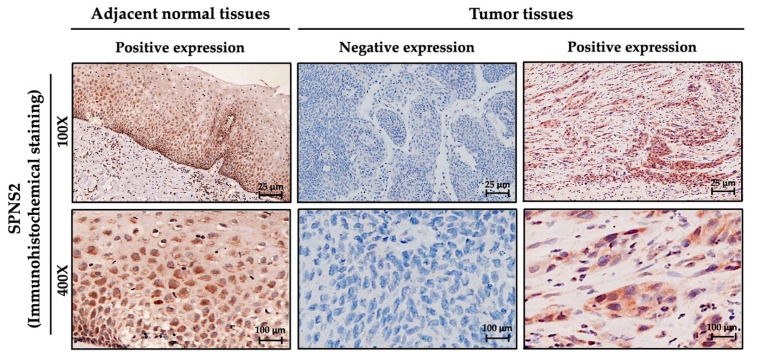
Overexpression of SPNS2 protein in primary OSCC tissues as detected by immunohistochemical staining. Representative immunohistochemical staining of SPNS2 protein indicating negative or positive cytoplasmic expression: (**left**) Normal tissue from adjacent to the tumor, and (**right**) Primary tumor tissue. Magnification 100× (**top**) and 400× (**bottom**). Scale bars: 25 μm (**top**) and 100 μm (**bottom**).

Table 1 lists the demographic and clinicopathologic characteristics of the OSCC patients in this study, including the cytoplasmic staining of SPNS2, gender, age, T (Tumor size), N (Lymph node), M (Metastasis), AJCC cancer stage, histological grade, and clinical therapy. Adjacent normal tissue from OSCC patients was used as a control. Among the 264 OSCC patients, 250 (94.7%) were males ranging in age from 31 to 88, with a mean age of 55.3 and median age of 54.0. Cytoplasmic staining identified 141 cases (53.4%) negative for SPNS2 and 123 cases (46.6%) positive for SPNS2, as shown in Figure 1. Tumor size (T) distribution was as follows: I (65 cases; 24.6%), II (81 cases; 30.7%), III (21 cases; 8.0%), and IV (97 cases; 36.7%). N (Lymph node) included 169 (64.0%) cases of N0 and 95 (36.0%) cases of N1. In terms of metastasis (M), 262 cases (99.2%) were M0 and 2 cases (0.8%) were M1. AJCC cancer stage distribution was as follows: stage I (50 cases; 18.9%), stage II (55 cases; 20.8%), stage III (31 cases; 11.7%), and stage IV (128 cases; 48.5%). In terms of histological grade, 42 cases (15.9%) were identified as well-differentiated (Well), 214 cases (81.1%) were moderately differentiated (Moderate), and 8 cases (3.0%) were poorly differentiated (Poor). A total of 162 patients (71.4%) received radiotherapy, and 65 patients (28.6%) received chemotherapy.

### 3.2. Correlations between SPNS2 Expression and Clinicopathologic Variables

Fisher’s exact test or Chi-square test was used to analyze the relationship between OSCC protein expression and clinicopathologic variables in order to determine the clinical significance of SPNS2 protein expression in 264 OSCC patients. Table 2 lists the correlation between SPNS2 protein expression and clinicopathological variables in the OSCC patients, with the research cohort divided into subgroups depending on whether they tested negative or positive for cytoplasmic SPNS2 expression. Cytoplasmic SPNS2 expression was correlated with T status (*p* = 0.026) and stage (*p* = 0.011); however, no correlation was observed between SPNS2 expression and age, gender, histological grade, lymph node metastasis, distant metastasis, smoking, chewing betel nut, or survival (*p* > 0.05) (Table 2).

### 3.3. Negative Cytoplasmic SPNS2 Expression was Associated with Short Overall Survival of Patients with OSCC Stage III/IV 

**Figure 2:**
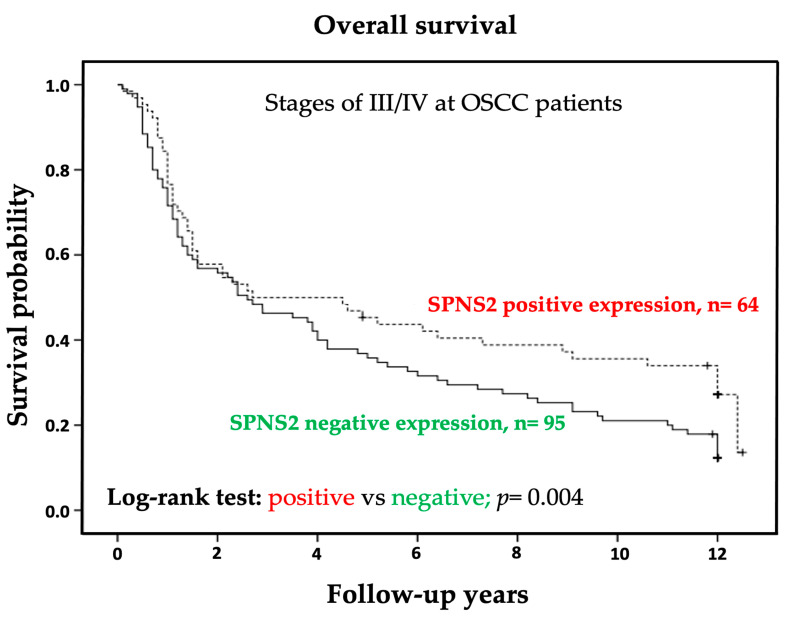
Kaplan–Meier survival analysis of negative and positive cytoplasmic SPNS2 protein expression in stage III/IV OSCC patients for use in log-rank tests of homogeneity using Kaplan–Meier curves. * *p* < 0.05.

We examined the correlation between cytoplasmic SPNS2 protein expression and overall survival of patients with OSCC stage III/IV using the Kaplan–Meier method and log-rank test to plot survival curves. Our results indicated that the overall survival time of the 95 OSCC stage III/IV patients who tested negative for SPNS2 protein expression was shorter than that of the 64 patients who tested positive (*p* = 0.004) (Figure 2).

### 3.4. Cox Proportional Hazard Model Analysis to Identify Prognostic Indicators in OSCC Patients

Cox proportional hazard models identified cytoplasmic SPNS2 protein expression as an independent prognostic indicator of overall survival in 159 OSCC stage III/IV patients. Univariate analysis identified SPNS2 expression, lymph node metastasis and histological grade as prognostic indicators of overall survival. Multivariate analysis identified SPNS2 expression, T status, lymph node metastasis and histological grade as independent prognostic indicators of the overall survival of OSCC stage III/IV patients (Table 3).

## 4. Discussion

The cause of oral cancers can be traced back to genetic as well as environmental factors. OSCC has been linked to genetic changes, such as alterations in chromosomes 3, 9, 11, and 13 [42]. A number of tumor markers have been used for the clinical diagnosis of OSCC; however, the high degree of tumor heterogeneity and complexity of underlying mechanisms mean that these biomarkers lack sufficient sensitivity and specificity to assess the prognosis of OSCC patients [43,44]. Previous literatures have reported that the aberrant expression of various oncogenes and tumor suppressor genes can have anti-tumor effects or tumor-promoting effects [45]. Various biomarkers have been associated with the incidence of OSCC with patients and disease progression, indicating their key role in tumorigenesis. We investigated the expression level of SPNS2 in OSCC and examined the prognosis of the patients.

The up-regulation or down-regulation of SPNS2 has been linked to many cancers, including acute myeloid leukemia, lung cancer, and colorectal cancer [35,37,38]. In the current study, immunochemical staining was used to detect SPNS2 expression in OSCC tissue. Results showed that positive staining of cytoplasmic SPNS2 protein expression was harder to detect in OSCC tissues, but can be more easily detected in normal tissues. Furthermore, the rate of SPNS2 low cytoplasmic expression in OSCC was significantly greater than those in normal control (Figure 1). Bradley et al. reported that SPNS2 mRNA levels were significantly lower in tissue samples from lung cancer patients (Stages 2B and 3) than in corresponding tissue from normal controls [35]. Thus, we theorized that SPNS2 could be a potential marker for the diagnosis of OSCC. We found that low cytoplasmic SPNS2 protein expression was correlated with T status and stage (Table 2). Consistent with our results, previous studies have implicated SPNS2 in the development of OSCC and tumor progression [35]. 

No previous study demonstrated any correlation between expression of SPNS2 and clinical outcome as well as survival in OSCC patients. Nonetheless, the prognostic value of SPNS2 protein expression in OSCC patients remains unknown. In our study, we found that the overall survival time of OSCC patients (stage III/IV) who tested negative for SPNS2 protein expression was shorter than that of patients who tested positive for SPNS2 (Figure 2). The results show the significance by using SPNS2 as a predictive marker of response. These observations may further support the involvement of SPNS2 in the T status and stage of OSCC (Table 2), and may play a decisive role in the overall treatment outcome of OSCC. However, Huang et al. reported that event-free survival and overall survival rates were significantly lower in acute myeloid leukemia patients with high SPNS2 expression than in patients with low SPNS2 expression. SPNS2 was also an independent dismal prognosis factor in chemotherapy and all-HSCT groups for event-free survival and overall survival using multivariate analysis. However, lacking of SPNS2 can reduce the ability of S1P to regulate lymphocyte transport, which leads to a decreased lymphocyte circulation in the tissue and increased in the proportion of T cells or NK cells, thereby killing tumor cells more effectively. On the other hand, SPNS2 can also promote tumor growth by transporting S1P to the extracellular environment [38]. In the current study, univariate and multivariate analysis both identified SPNS2 expression, T status, lymph node metastasis, and histological grade as important independent prognosis factors affecting the overall survival of OSCC stage III/IV patients (Table 3). Overall, this study determined that SPNS2 protein may be a useful prognostic marker for OSCC patients and a potential therapeutic target for OSCC treatment. 

Our results indicate that SPNS2 may have tumor suppressor function in OSCC cells, which is consistent with previous reports [46,47,48]. In lung cancer cells, the overexpression of SPNS2 has been shown to induce cell apoptosis, and SPNS2 knockdown enhances cell migration. Inhibiting S1P synthesis can eliminate the effects of SPNS2 knockdown on cell migration. Gu et al. reported that SPNS2 expression impairs pro-survival pathways mediated by glycogen synthase kinase-3β (GSK-3β) and signal transducer and activator of transcription 3 (STAT3). These results provide evidence that SPNS2 plays a key role in regulating the cellular functions of lung cancer cells and that SPNS2 down-regulation is a potential risk factor for lung cancer [35]. In colon cancer cells, SPNS2 has been found to activate the AKT and ERK pathways [37]. Sphingolipid transporter 3 (SPNS3) mainly participates in the Sphingolipid signaling pathway and SPNS3 may also develop its function through a similar mechanism as SPNS2 from the KEGG gene set enrichment data in AML [49]. SPNS3 can mediate the process of mammalian cell apoptosis and autophagy [50,51,52]. Certain genetic variations in the autophagy-lysosome pathway play a crucial role in cancer development [53]. 

This study recruited 264 OSCC patients from January 2000 to December 2008, the classifications of these patients were based on the AJCC, 7th Edition of the TNM classification. After 2019, the classifications of OSCC new patients follows the AJCC, 8th Edition of the TNM classifications. Currently, the study mentioned above is restricted by the IRB. Thus, the relevant information of these patients has been decoded, and the identifiable patient data have also been deleted. Therefore, it is difficult to classify old samples again according to the AJCC, 8th Edition of the TNM classification. Based on these limitations, our results may be slightly different from those based on the AJCC, 8th Edition of the TNM classification, and we will include these differences in the guidelines of our research team. On the other hand, in order to explore the potential functions and mechanisms of SPNS2 and its relationship with therapeutic drugs, it is necessary to further analyze the tumor suppressor function of SPNS2 in OSCC based on in vitro and in vivo experiments. 

In conclusion, we determined that SPNS2 is correlated with T status and stage of tumors in OSCC patients. We also identified a link between SPNS2 expression and overall survival of stage III/IV OSCC patients. Kaplan–Meier analysis revealed that negative cytoplasmic SPNS2 protein expression was associated with shorter overall survival rate, which suggests that SPNS2 could potentially be used as a prognostic indicator for patients with OSCC and a potential therapeutic target for OSCC treatment. 

## Figures and Tables

**Table 1:** Demographics and characteristics of patients with oral squamous cell carcinoma.

Factors	(*n* = 264)	Percentage
**Cytoplasmic staining of SPNS2**		
Negative	141	53.40%
Positive	123	46.60%
**Gender**		
Male	250	94.70%
Female	14	5.30%
**Age (Year)**		
Range	31–88	
Mean	55.3	
Medium	54	
**T (Tumor size)**		
I	65	24.60%
II	81	30.70%
III	21	8.00%
IV	97	36.70%
**N (Lymph node)**		
N0	169	64.00%
N1	95	36.00%
**M (Metastasis)**		
M0	262	99.20%
M1	2	0.80%
**AJCC cancer stage**		
I	50	18.90%
II	55	20.80%
III	31	11.70%
IV	128	48.50%
**Histological grade**		
WD	42	15.90%
MD	214	81.10%
PD	8	3.00%
**Clinical therapy**		
Radiotherapy	162	71.40%
Chemotherapy	65	28.60%

WD: Well differentiated; MD: moderately differentiated; PD: poorly differentiated. AJCC: American Joint Committee on Cancer.

**Table 2:** Clinicopathologic variables correlated with SPNS2 expression in patients with oral squamous cell carcinoma.

Cytoplasmic Staining of SPNS2				
Variables	Negative	Positive	(*n* = 264)	*p*-Value ^a^
**Age**	55.7 ± 10.9	55.3 ± 11.2		0.803
**Gender**				
Male	136 (96.5%)	114 (92.7%)	250	0.173
Female	5 (3.5%)	9 (7.3%)	14	
**Histological grade**				
WD	23 (16.3%)	19 (15.4%)	42	0.848
MD/PD	118 (83.7%)	104 (86.4%)	222	
**T status**				
T1/T2	69 (48.9%)	77 (62.6%)	146	0.026 *
T3/T4	72 (51.1%)	46 (37.4%)	118	
**Lymph node metastasis**				
No	86 (61.0%)	83 (67.5%)	169	0.273
Yes	55 (39.0%)	40 (32.5%)	95	
**Distant metastasis**				
M0	139 (98.6%)	121 (100%)	262	0.501
M1	2 (1.4%)	0 (0%)	2	
**Stage**				
I, II	46 (32.6%)	59 (48.0%)	105	0.011 *
III, IV	95 (67.4%)	64 (52.0%)	159	
**Smoking**				
No	34 (33.3%)	34 (41.0%)	68	0.284
Yes	68 (66.7%)	49 (59.0%)	117	
**Betel nut chewing**				
No	30 (50.0%)	29 (50.9%)	59	0.924
Yes	30 (50.0%)	28 (49.1%)	58	
**Survival**				
≤3 year	57 (40.4%)	51 (41.5%)	108	0.865
>3 year	84 (59.6%)	72 (58.5%)	156	
≤5 year	71 (50.4%)	60 (48.8%)	131	0.799
>5 year	70 (49.6%)	63 (51.2%)	133	

WD: Well differentiated; MD: moderately differentiated; PD: poorly differentiated. M0: No distant metastasis; M1: distant metastasis. ^a^ The *p*-value using Fisher’s exact test or Chi-square test. * *p* < 0.05.

**Table 3:** Overall survival of III/IV stage and clinicopathologic variables of patients with oral squamous cell carcinoma using univariate and multivariate analysis.

	Univariate Analysis			Multivariate Analysis		
Variables (*n* = 159)	Hazard Ratio ^a^	95% CI	*p*-Value	Hazard Ratio ^a^	95% CI	*p*-Value
**Expression of SPNS2**						
Positive	1			1		
Negative	1.44	1.001–2.063	0.049 *	1.45	1.001–2.083	0.049 *
**T status**						
T1, T2	1			1		
T3, T4	1.1	0.761–1.577	0.624	1.68	1.063–2.660	0.026 *
**Lymph node metastasis**						
No	1			1		
Yes	1.53	1.095–2.137	0.013 *	1.73	1.153–2.594	0.008 *
**Distant metastasis**						
M0	1			1.04		
M1	1.32	0.486–3.557	0.590	1	0.231–4.001	0.957
**Histological grade**						
WD	1			1		
MD, PD	1.99	1.163–3.402	0.012 *	1.77	1.017–3.066	0.043 *

WD: Well differentiated; MD: moderately differentiated; PD: poorly differentiated. M0: No distant metastasis; M1: distant metastasis. 95% CI: 95% Confidence interval; ^a^ Hazard ratio was adjusted for gender and age. * *p* < 0.05.

## Data Availability

The data used and analyzed during the present study are available from the corresponding author upon reasonable request. The data are not publicly available due to possible personal information breaches though they were de-linked.
